# A Case of Congenital Pulmonary Vein Stenosis with Secondary Post-Capillary Pulmonary Hypertension and Left Sided Congestive Heart Failure in a Cat

**DOI:** 10.3390/vetsci10010023

**Published:** 2022-12-29

**Authors:** Karin Kriström, Erika Karlstam, Tove Nielsen, Anne Lagerqvist, Mark Dirven

**Affiliations:** 1 Evidensia Södra Djursjukhuset, 14175 Stockholm, Sweden; 2 Department of Pathology and Wildlife Diseases, National Veterinary Institute, 75189 Uppsala, Sweden; 3 Anicura Djursjukhuset Albano, 18236 Danderyd, Sweden; 4 Anicura Jönköping Djursjukhus, 55475 Jönköping, Sweden

**Keywords:** pulmonary veins, stenosis, congenital heart disease, feline

## Abstract

**Simple Summary:**

Left-sided congestive heart failure and post-capillary pulmonary hypertension are commonly associated with left atrial enlargement. An instrumental step in diagnosing left-sided congestive heart in cats is confirming the presence of left atrial enlargement. However, in selected cases, left-sided congestive heart and post-capillary pulmonary hypertension may be present while the left atrium is not enlarged. This case report presents a 5-month-old Maine Coon cat with congenital pulmonary vein stenosis resulting in severe left-sided congestive heart and post-capillary pulmonary hypertension without left atrial enlargement.

**Abstract:**

A five-month-old, 3.8 kg intact male Maine coon cat presented for dyspnea characterized by increased respiratory effort in addition to open-mouth breathing. Thoracic radiographs showed pectus excavatum, enlarged cardiac silhouette, and generalized interstitial patterns. Echocardiography revealed normal left atrial (LA) and left ventricular dimensions. A large tubular structure, suspected to be a distended pulmonary vein (PV), was identified as draining into the LA. Severe eccentric and concentric right ventricular hypertrophy and paradoxical septal motion were noted. Based on Doppler echocardiography, both pulmonary venous and pulmonary artery pressure was severely elevated. Clinical, radiographic, and echocardiographic abnormalities were hypothesized to result from pulmonary vein stenosis (PVS), causing severely elevated pulmonary venous pressures and resulting in clinical signs of left-sided congestive heart failure (L-CHF) and severe post-capillary pulmonary hypertension (Pc-PH). The prognosis for good quality of life was assessed as poor, and the owner elected euthanasia. Necropsy confirmed the presence of PVS with severe dilation of the PVs draining all but the left cranial lung lobe. All lung lobes except the left cranial lobe had increased tissue density and a mottled cut surface. This case report shows that, in rare cases, both L-CHF and Pc-PH may be present without LA enlargement. To the authors’ knowledge, this is the first report on PVS in veterinary medicine.

## 1. Case Description

A five-month-old, 3.8 kg male Maine Coon cat was presented with signs of dyspnea characterized by increased respiratory effort in addition to open-mouth breathing. Clinical signs had been progressive and present for at least two months.

At admission, the cat was alert and responsive but showed an abdominal breathing pattern, increased respiratory rate (60/min), and cyanotic oral mucous membranes. Body condition score was evaluated to be normal (4–5/9), and body temperature was 37.3 °C. Heart rate was normal for a cat for a cat in hospital environments (210 bpm), and heart rhythm was regular. Auscultation revealed a parasternal systolic heart murmur with moderate intensity on the left hemithorax. In contrast, heart sounds were muffled on the right hemithorax.

**Figure 1:**
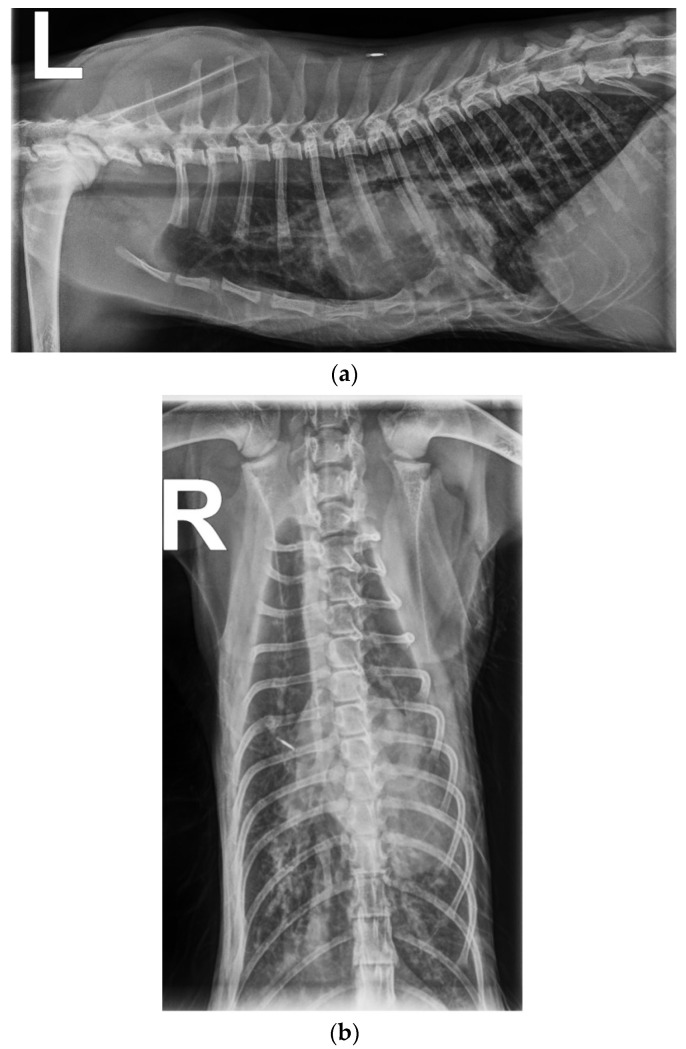
(**a**,**b**) Left lateral thoracic radiographs showing a generalized, unstructured interstitial pattern. The cat also had pectus excavatum, and the caudal sternum was dorsally deviating, displacing the cardiac silhouette to the left.

Survey thoracic radiographs showed that the cat had pectus excavatum and the caudal sternum was dorsally deviating, displacing the cardiac silhouette to the left. This made it challenging to assess size and shape of the cardiac silhouette reliably. The pulmonary parenchyma had a generalized increased unstructured interstitial pattern, moderate in the caudal part and mild in the cranial part of the lung. The interpretation of the lung parenchyma was complicated by wet fur. The borders of the pulmonary vessels could not be adequately evaluated due to the increased opacity in the lung (Figure 1a,b).

Transthoracic echocardiography was performed according to published guidelines [1] using an ultrasound unit (GE, Logiq E9, Milwaukee, WI, USA) equipped with 6 to 12 MHz phased-array transducers. The cat’s abnormal thoracic anatomy and clinical condition did not allow for standard windows and views. The echocardiographic examination was subsequently performed in a modified left apical window. A simultaneous electrocardiogram tracing was recorded during the echocardiographic examination. The pectus excavatum anomaly and the subsequent displacement of the heart limited accessible echocardiographic views. No right parasternal transthoracic views could be obtained, and all evaluations were made from left apical- and left cranial views.

**Figure 2:**
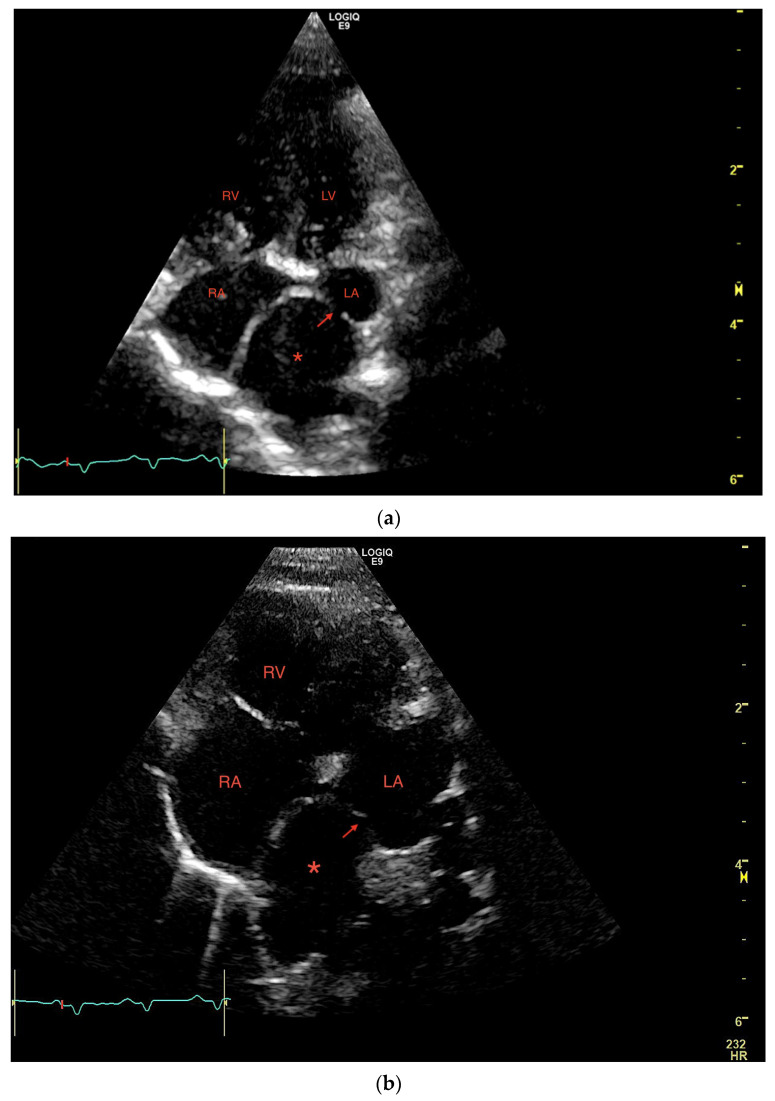
(**a**,**b**) Transthoracic left apical window modified 4-chamber optimized for showing the dilated PV (red *) opening into the LA through a narrow ostium at the PV-LA junction (red arrow). LA: left atrium, PV: pulmonary vein, RA: right atrium, RV: right ventricle.

M-mode and two-dimensional (2D) examinations showed normal left ventricular size and function, normal left ventricular outflow tract, and normal left atrial (LA) size. An abnormal tubular structure with a diameter of approximately 12 mm was visualized in direct proximity to the LA base, with a suspicion of a small opening (1–2 mm) connecting this structure to the LA (Figure 2a,b).

The right ventricle showed both eccentric and concentric hypertrophy, and paradoxical interventricular septal motion, all indicating elevated right ventricular systolic pressures. The right atrium was subjectively assessed to be normal in size, and the main pulmonary artery was subjectively assessed to be dilated.

**Figure 3:**
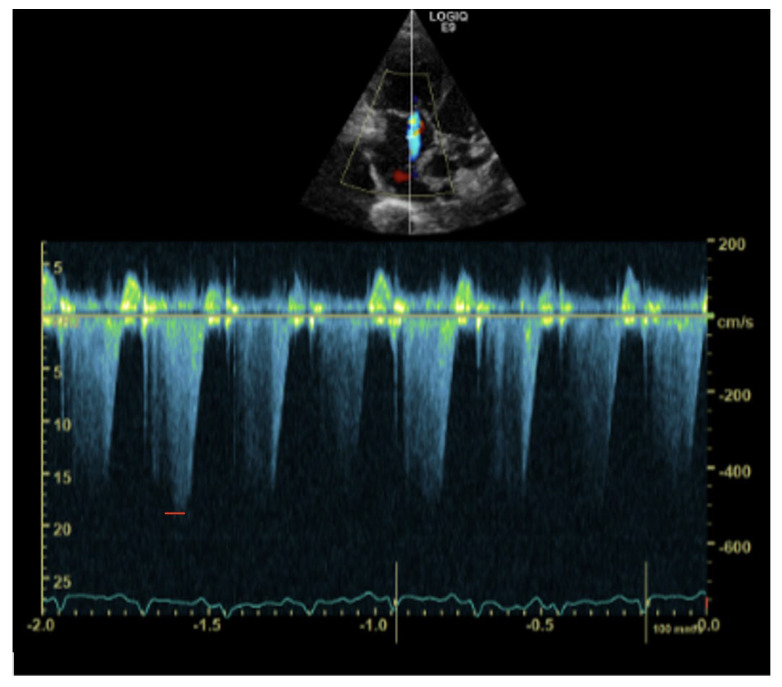
Transthoracic left apical window modified 4–chamber optimized for the right heart. Doppler imaging shows a tricuspid regurgitation with a peak blood flow velocity of 5 m/s (red stripe), representing an estimated RV pressure of at least 100 mmHg.

Doppler imaging revealed a tricuspid regurgitant jet, with a peak blood flow velocity of 5 m/s, suggesting severely elevated right ventricular systolic pressure (estimated pressure at least 100 mm Hg) (Figure 3).

**Figure 4:**
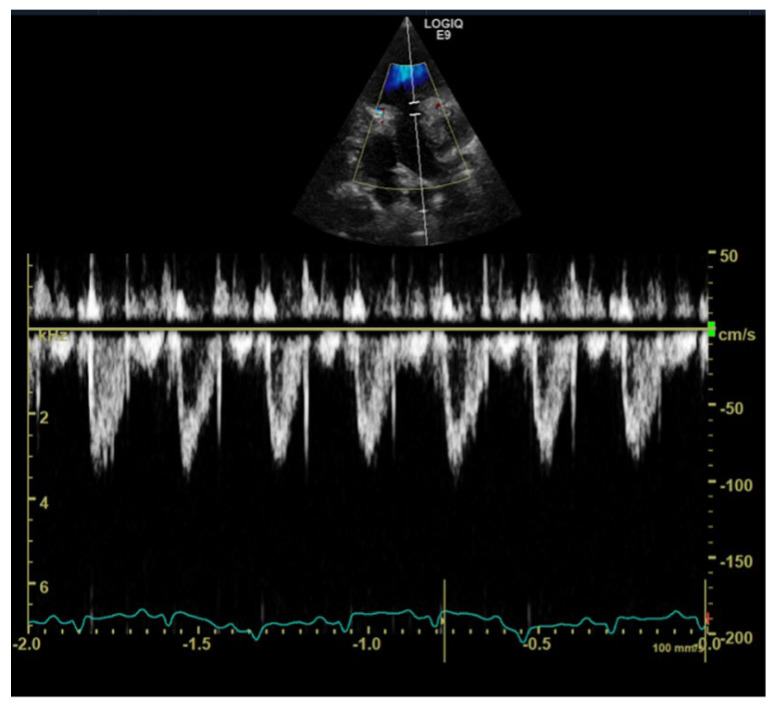
Transthoracic left apical window modified 4–chamber view optimized for the right ventricular outflow tract. Doppler (color and spectral) examination shows a pulmonary artery profile with type II profile but normal peak blood flow velocity, suggesting increased pulmonary artery pressures.

The pulmonary artery velocity was within normal reference ranges (0.9 m/s; estimated pressure 3.2 mmHg). Pulsed wave spectral Doppler examination of pulmonary outflow showed a Type II appearance of the Doppler profile, characterized by shortened acceleration time. Type II profile has been associated with high pulmonary pressure or vas-cular resistance in humans and dogs [2] (Figure 4). There were no signs of right or left ventricular outflow tract obstruction nor intracardiac shunts.

**Figure 5:**
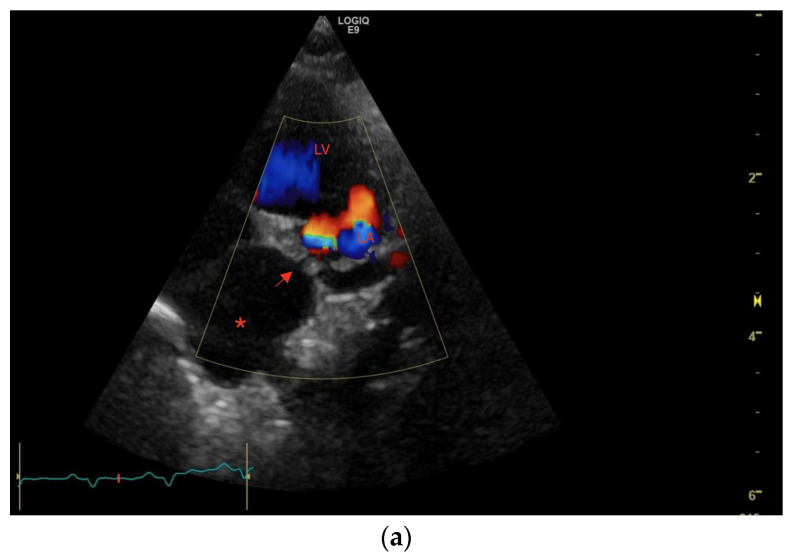

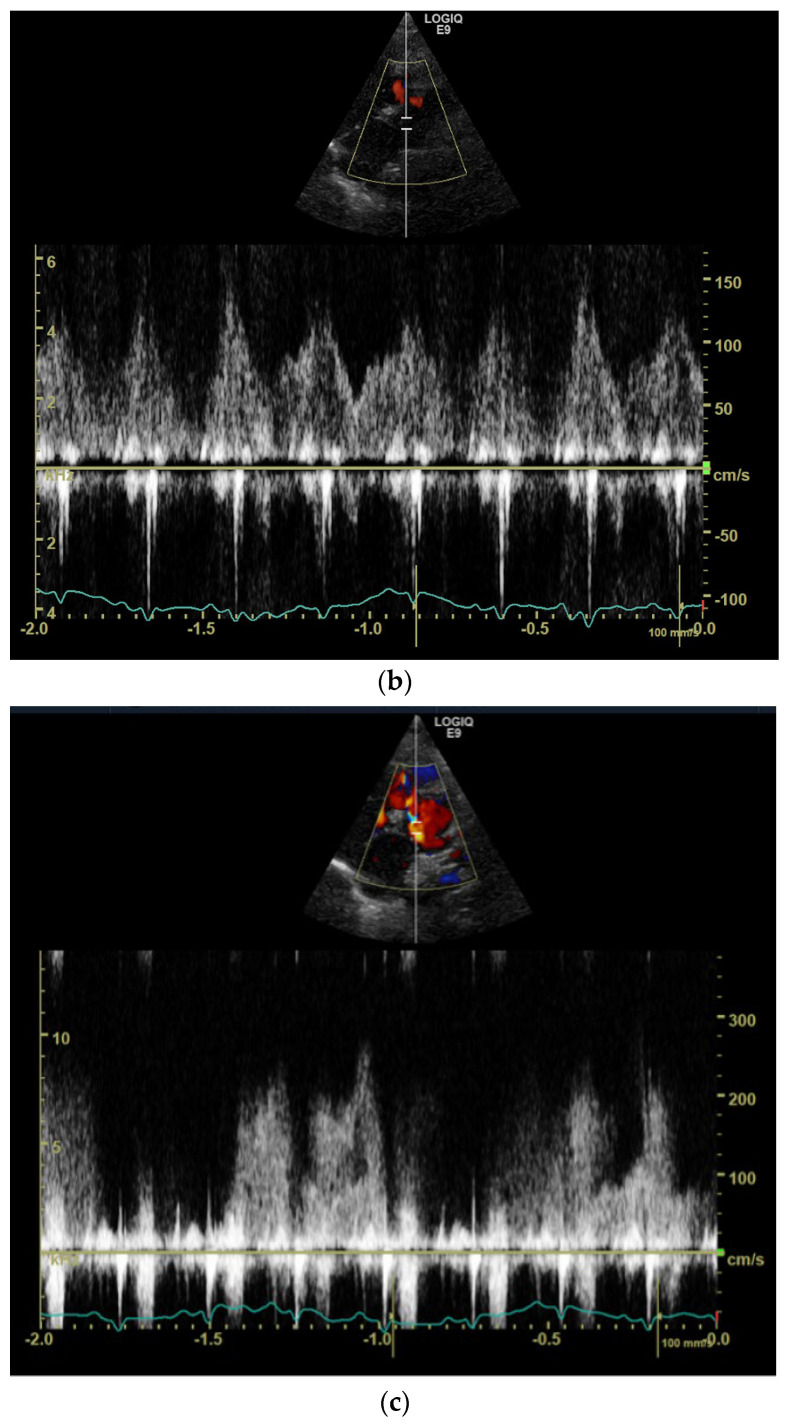
(**a**–**c**) Transthoracic left apical window modified 4–chamber optimized for showing the dilated PV (red *) emptying into the LA through a narrow ostium at the PV-LA junction. The anatomy and clinical condition of the cat did not allow for standard windows and views. (**a**) Color Doppler examination showing a turbulent blood flow between the dilated PV (red arrow) and the LA.; (**b**) Spectral Doppler examination performed with pulsed wave gate placed at what was believed to be the PV inlet. This view shows a continuous flow in at least one cycle. The angle is suboptimal, and therefore velocity is underestimated in the image.; (**c**) Spectral Doppler examination performed with pulsed wave gate placed at what was believed to be the PV inlet. The image shows a peak blood flow velocity of 2.6 m/s representing an estimated pressure gradient of 27 mmHg between the dilated PV and the LA and, therefore, an estimated PV pressure of at least 32 mmHg. The angle is suboptimal, and therefore continuous flow could not be shown. LA: left atrium, PV: pulmonary vein.

One normal PV could be identified as draining into the LA via an ostium in the left LA wall. The abnormal tubular structure identified near the LA was confirmed to drain into the LA through a narrow opening with a continuous blood flow and a peak blood flow velocity of 2.6 m/s, corresponding to an estimated pressure difference between the structure and the LA of 27 mmHg (Figure 5a,b). The structure was interpreted as a distended PV emptying into the LA through a stenotic ostium, and the high blood flow velocity suggested severely elevated pulmonary venous pressures.

The owner declined further treatment, and the cat was euthanized. The owner consented to post-mortem examination, and the cat was sent for pathology.

**Figure 6:**
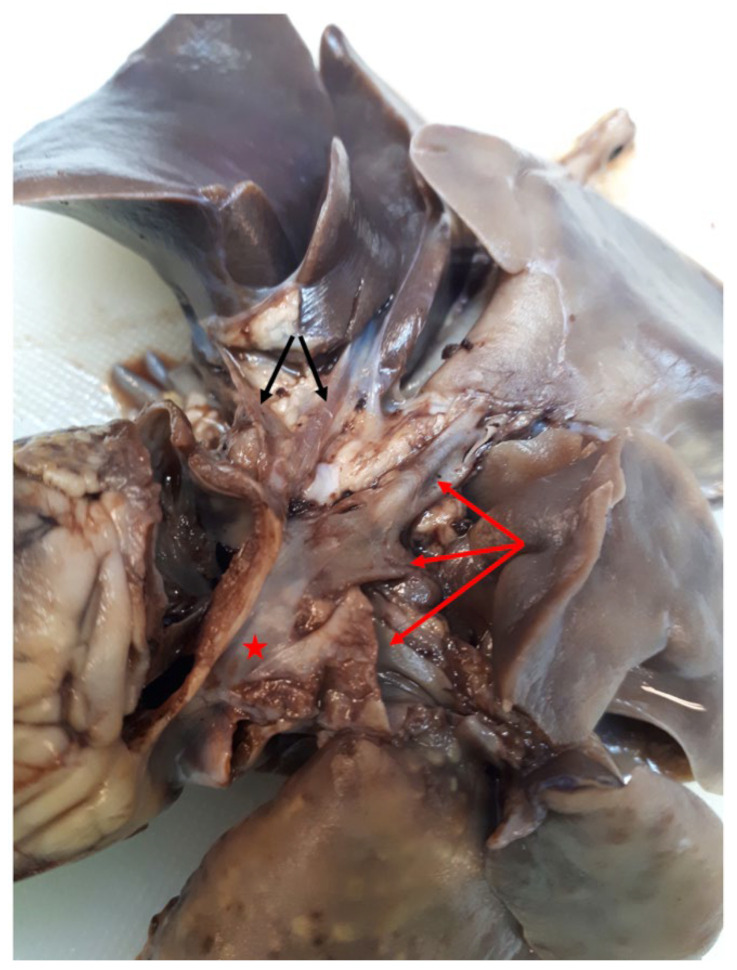
Necropsy image showing the anomalous junction of the pulmonary veins into the LA. Two normal PVs are seen, draining the cranial and caudal part of the left cranial lobe (marked with black arrows), opening in a normal ostium in the LA left wall. Three severely dilated PVs draining other lung lobes (red arrows) merge into a sac-like, dilated formation located in direct proximity to the LA (red star). LA:left atrium, PV: pulmonary vein.

Necropsy revealed moderate eccentric and concentric hypertrophy of the right ventricle and a moderately enlarged right atrium. The size of the left ventricle and atrium was within normal variation. The mitral, tricuspid, aortic, and pulmonary valves were normal. Two normal PVs draining the cranial and caudal part of the left cranial lung lobe were identified as draining into a common ostium in the posterior LA wall. The PVs draining the left caudal and the accessory lobe were severely dilated and merged into a sac-like formation at the LA. The PVs draining the right cranial, right intermediate, and right caudal lobes were moderately dilated and merged into the same sac-like formation (Figure 6). A severely stenotic ostium could be identified from the atrial side, whereas no opening could be identified from the pulmonary side of the sac-like formation.

The cranial and caudal parts of the left cranial lobe had a relatively normal macroscopic appearance. All other lung lobes were voluminous and had increased tissue density and multiple 2–3 mm yellow-white nodules/plaques on the surface. Microscopical examination of the lungs showed a pronounced chronic pulmonary edema in the lung lobes drained by the dilated, stenotic lung veins. In the left cranial lung lobe, drained by normal pulmonary veins, the edema was assessed as mostly acute. Moreover, in a subset of pulmonary vessels, mostly veins and smaller vessels, mild to moderate mural necrosis and inflammation was seen.

## 2. Discussion

Pulmonary vein stenosis is a rare anomaly that, to our knowledge, has not been reported in veterinary medicine. The condition may be congenital [2,3,4] or acquired secondary to radiofrequency ablation or other surgical interventions, which have been reported in humans and experimental dog models [5,6,7].

During early gestation, the heart and the lungs develop separately from the splanchnic mesoderm and primitive foregut. Only later, during the septation stage, a primitive common PV starts developing in the form of small bulges from both the splanchnic mesoderm and the primitive LA. The two separate portions of primitive PV later join, and the primitive common PV begins to drain blood from the pulmonary system. This primitive common PV further develops into several individual PV, draining the different lung lobes [2,8,9]. A failure to incorporate the primitive common PV into the LA may lead to various congenital abnormalities, of which cor triatriatum sinister (CTS), PVS, and partial or total anomalous pulmonary venous return are the most common [2,10,11,12].

Echocardiographic diagnostics may be challenging as PVS has many clinical and diagnostic similarities with other stenotic lesions in or proximal to the LA, such as CTS and supravalvular mitral valve stenosis (SMS). Supravalvular mitral stenosis may, however, be differentiated by localizing the obstructive membrane relative to the LA appendage (LAA) and the localization of the PVs [4,13]. In SMS, the obstructive membrane is located apical to the LAA, resulting in a basal dilated chamber, including the LAA and the basal part of the LA, into which, all PVs empty [4,13]. In CTS, the obstructive membrane is located basal to the LAA, resulting in a non-dilated chamber consisting of the LAA and the apical part of the LA, and a dilated chamber consisting of the basal part of the LA into which the PVs empty [12]. In SMS and in classic CTS, all PVs drain into the basal dilated chamber via non-obstructed and normally located PV ostia [13]. In contrast, in cases of PVS, the obstruction is located at the PV ostium, and the dilated chamber consists of the obstructed PV(s) trying to empty into the LA via the stenotic ostium. Unaffected PVs drain into the LA without the obstruction of blood flow [2].

**Figure 7:**
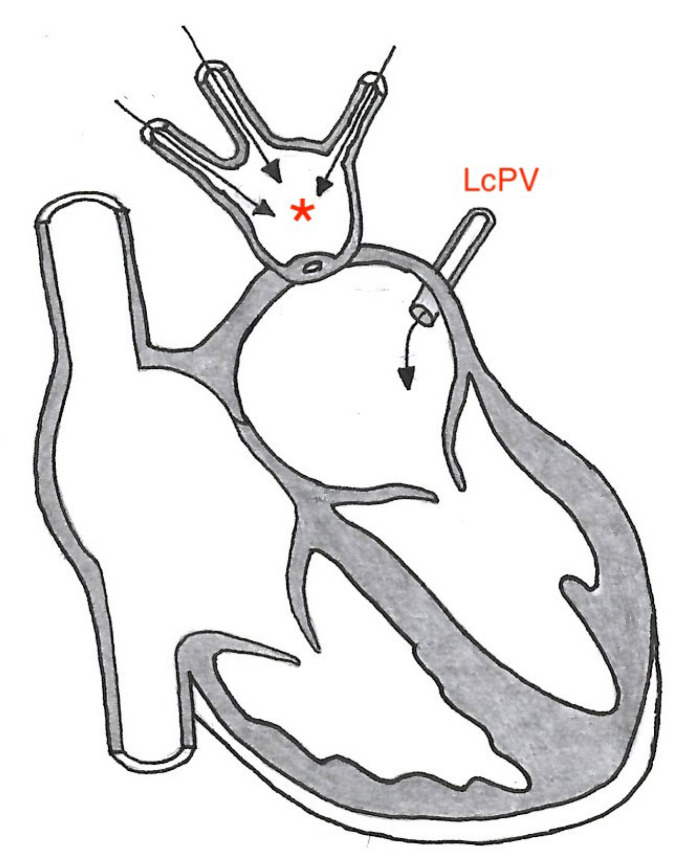
Diagram showing the collecting dilated structure attached to the LA (red *) and the PVs draining the left caudal, accessory, right caudal, and the right cranial lung lobe emptying into the dilated common structure. The normal PV draining the left cranial lung lobe (LcPV) empty via the ostium in the left LA-wall. LA: left atrium, LcPV: Left cranial lung lobe, PV: pulmonary vein.

Subtotal CTS is a variant of CTS where the PVS of one side of the lungs assemble in a common collecting structure proximal to the PV/LA junction, and the PVS of the other side of the lungs drain normally into the LA [4,14,15]. This anomaly may be challenging to differentiate from PVS due to similarities on both echocardiography and on necropsy. In the present case only the cranial left lung lobe was drained through a normal PV into the LA whereas the PV of the left caudal lung lobe drained, together with the PVs of the right side of the lung, into the common collecting structure, confirming our suspicion of severe PVS (Figure 7).

Clinical signs of PVS in humans usually presents early in life, are often unspecific and resemble those seen with any lesion that results in increased pulmonary venous pressures and L-CHF [3,10,11].Thoracic radiographs usually demonstrate diffuse or localized interstitial and alveolar patterns indicative of L-CHF and sometimes pleural effusion [11]. An instrumental step in diagnosing L-CHF in cats is confirming the presence of LA enlargement, which in emergency settings often is performed with point-of-care ultrasound [16,17]. The absence of LA enlargement in cases of PVS may make it challenging to establish a diagnosis, and in some cases, multiple diagnostic modalities may be required. In humans, an echocardiographic finding of turbulent PV flow with an increased flow velocity (>1.1–1.5 m/s) is considered a reliable index of a PVS diagnosis [6,11]. The maximal flow velocity over the stenotic ostium in our cat was at least 2.6 m/s, supporting our diagnosis of severe PVS. Other recommended diagnostic modalities are ECG-gated computed tomography angiography, nuclear medicine lung perfusion imaging, and cardiac magnetic resonance [11,18]. However, using these modalities may be challenging in veterinary medicine due to the need for anesthesia in already compromised animals.

The anatomy of the obstruction in PVS varies and studies from human medicine report changes ranging from complete or partially obstructive diaphragm, a stenotic segment, to complete atresia of a vein. Many individuals may have different obstructions in different PVs [3,10]. A common histological finding in congenital PVS in humans is an overgrowth of connective tissue with medial hypertrophy and intimal fibrosis at the pulmonary venous-LA junction [19,20]. The stenotic lesion found at necropsy in the present case was severe and almost no opening could be identified at the LA/PV junction. No obstructive membrane or local overgrowth of connective tissue/medial hypertrophy could be identified.

The obstruction increases pulmonary venous hydrostatic pressure in the lung lobes drained by the affected PV(s). In severe cases, chronic pulmonary venous pressure elevations lead to secondary vascular remodeling, and increased pulmonary vascular resistance that may result in post-capillary PH development. The high hydrostatic venous pressure may also spread retrogradely to the pulmonary capillaries and lead to secondary pulmonary edema, where the resulting hypoxia worsens PH through pulmonary vascular constriction [10,20].

Pulmonary hypertension (PH) is defined as excessively high pressure in the pulmonary vasculature and is caused by increased pulmonary vasculature resistance, increased pulmonary blood flow, increased pulmonary venous pressure, or a combination thereof [21,22,23]. The condition is rarely reported in cats, and while most underlying mechanisms appear similar to those reported in humans and dogs, others may differ [23]. The most reported conditions associated with PH in cats are congenital heart disease with a left to right shunting of blood, pulmonary thromboembolism, pulmonary fibrosis, chronic upper airway obstruction, and infections with lung- and heartworms [23,24,25,26,27]. The cat in the present case did not have any signs of other congenital heart disease than PVS to explain PH on echocardiography. Other potential causes of PH could not be actively excluded antemortem due to the cat’s clinical condition. Postmortem examination did, however, not reveal any additional pathological findings that could be associated with PH development.

Pectus excavatum is an unusual defect of the thoracic skeleton defined by a dorsal deviation of the caudal sternebrae. The exact etiology remains unknown, although possible underlying causes are embryonic sternocostal growth disturbance and connective tissue disorders [28,29,30,31]. Common clinical signs of pectus excavatum are failure to thrive, respiratory distress, exercise intolerance, coughing, cardiac murmur, and cyanosis, depending on the severity of the lesion [29,30,31]. Severe pectus excavatum may also cause mechanical compression of the PVs, leading to a variant of PVS [32,33]. A possible influence on breathing from the malformation cannot be completely ruled out in the present case. However, it was not assessed as the primary cause of the cats’ respiratory distress, which was further confirmed by pathology. The malformation did, however, result in heart displacement in the thorax, making the echocardiographic examination challenging.

To the authors’ best knowledge, there are no reported cases of PVS in veterinary medicine and hence no recommended treatment regimens. Diuretic treatment may relieve the acute clinical signs of L-CHF by reducing preload. This will, however, not decrease PV pressures in cases of severe PVS [34]. In human medicine, surgical treatment with balloon dilatations, stenting, or marsupialization of the stenosis has shown limited success due to restenosis. Recent reports from human medicine of surgical treatment with drug eluting stents have shown promising results [35]. The prognosis is, however, guarded despite adequate therapy in severe cases [19]. The severity and duration of clinical signs, indicative of a high risk for irreversible changes in the pulmonary vasculature, and the lack of effective treatment in cats resulted in our recommendation of euthanasia in this case.

## 3. Conclusions

This case report describes a Maine coon cat diagnosed with severe and most likely congenital PVS, secondary pc-PH, and L-CHF. The case shows that, in rare cases, both L-CHF and Pc-PH can be present without LA enlargement.

## Data Availability

The data presented in this study are available on request from the corresponding author. The data are not publicly available due to professional secrecy.
